# Melatonin Cytotoxicity Is Associated to Warburg Effect Inhibition in Ewing Sarcoma Cells

**DOI:** 10.1371/journal.pone.0135420

**Published:** 2015-08-07

**Authors:** Ana M. Sanchez-Sanchez, Isaac Antolin, Noelia Puente-Moncada, Santos Suarez, Marina Gomez-Lobo, Carmen Rodriguez, Vanesa Martin

**Affiliations:** 1 Departamento de Morfología y Biología Celular, Facultad de Medicina, c/Julian Claveria, 33006 Oviedo, University of Oviedo, Oviedo, Spain; 2 Instituto Universitario de Oncología del Principado de Asturias (IUOPA), Oviedo, Spain; Johns Hopkins University, UNITED STATES

## Abstract

Melatonin kills or inhibits the proliferation of different cancer cell types, and this is associated with an increase or a decrease in reactive oxygen species, respectively. Intracellular oxidants originate mainly from oxidative metabolism, and cancer cells frequently show alterations in this metabolic pathway, such as the Warburg effect (aerobic glycolysis). Thus, we hypothesized that melatonin could also regulate differentially oxidative metabolism in cells where it is cytotoxic (Ewing sarcoma cells) and in cells where it inhibits proliferation (chondrosarcoma cells). Ewing sarcoma cells but not chondrosarcoma cells showed a metabolic profile consistent with aerobic glycolysis, i.e. increased glucose uptake, LDH activity, lactate production and HIF-1α activation. Melatonin reversed Ewing sarcoma metabolic profile and this effect was associated with its cytotoxicity. The differential regulation of metabolism by melatonin could explain why the hormone is harmless for a wide spectrum of normal and only a few tumoral cells, while it kills specific tumor cell types.

## Introduction

Ewing sarcoma is the second most frequent primary bone tumor after osteosarcoma, accounting for 10–15% of these pathologies. It primarily affects children and young adults, with a peak incidence in the second decade of life. Nowadays, a combination of chemotherapy, surgery and radiation therapy results in 65% of patients free of disease after 4 years in those without metastases at diagnosis. However, even using the best combination of chemotherapy, surgery and radiation, 24–35% of patients still relapse, and this percentage is strikingly higher if patients had metastasis at diagnosis [1].

Antitumoral effects of melatonin have been extensively described in a wide variety of tumor cell types. This indolamine inhibits proliferation in the majority of tumor cells through several possible intracellular signaling pathways [2] including antioxidant actions [3–5]. However, melatonin is also able to kill some specific tumor types, such as Ewing sarcoma or hematological malignancies, by means of both the extrinsic and the intrinsic pathways of apoptosis [6,7]. Moreover, we have previously shown that melatonin not only kills Ewing sarcoma cells, but also shows synergy with vincristine, ifosfamide and other chemotherapeutic drugs currently used to treat this disorder [8,9].

Melatonin has been well characterized as a potent antioxidant, and its neuroprotective and antiproliferative effects are tightly associated with a decrease in reactive oxygen species (ROS) [4]. However, melatonin effects on tumor cells do not always correspond with an antioxidant effect. In fact, previous data obtained in our laboratory indicate that the cytotoxicity of melatonin in Ewing sarcoma cells is mediated by an increase in ROS [10]. Such increase in ROS has been also described in other tumor cells where melatonin cytotoxicity was also reported [11,12]. Many anticancer agents work by further increasing cellular levels of ROS, to overcome the antioxidant detoxification capacity of the cancer cell and induce cell death [13]. However, based on previous data, it seems clear that the fate of tumor cells following administration of melatonin is dependent on the intracellular redox state (antioxidant for antiproliferative effects vs. prooxidant for cytotoxic effects). But why the same molecule decreases intracellular oxidants in most normal and tumoral cells but increases free radicals in other specific types of tumors? Given that melatonin is an antioxidant in vitro [14], it is likely that its pro-oxidant effect in some tumors is indirect and due to intrinsic characteristics of specific tumor cells.

Energy metabolism accounts for the production of most intracellular ROS, and it is frequently altered in cancer [15,16]. The metabolic needs of highly proliferating tumor cells differ from normal cells, but also between different types of cancer [17]. Most tumor cells have an increased glucose uptake, allowing them to obtain higher amounts of pyruvate that is then used as an energy source, as it is converted to lactate in a process called aerobic glycolysis or Warburg effect [18]. This method of production of ATP is much less efficient than oxidative phosphorylation, but acceleration of glycolysis after increasing glucose uptake compensates for its inefficiency. Importantly, the contribution of the Warburg effect to energy metabolism is very marked in some tumors, but reduced or inexistent in others [19].

We hypothesized that melatonin could have different effects (antiproliferative vs cytotoxic) on tumoral cells depending on their intrinsic glycolytic metabolism. We show for the first time that melatonin regulates this metabolism, inhibiting the hallmarks of Warburg effect in Ewing sarcoma cells. Such inhibition is associated to the inactivation of HIF-1α, the main regulator of aerobic glycolysis, and to melatonin´s cytotoxicity.

## Material and Methods

### Cell culture and reagents

sw-1353 (chondrosarcoma) and A-673(Ewing sarcoma) cell lines were purchased from American Type Culture Collection (Teddington, United Kingdom) and TC-71 and A-4573 (Ewing sarcoma) cell line were a generous gift from Dr J.A. Toretsky (Departments of Oncology and Pediatrics, Georgetown University, Washington DC, USA). Cells were maintained at 37°C in a humidified atmosphere of 5% CO_2_, and subcultured once a week using a 0.25% trypsin solution. Cell culture reagents were purchased from Sigma (Sigma Chemical Co., St Louis, MO, USA) except for fetal bovine serum (FBS), which was purchased from Gibco (Invitrogen Life Technologies, Barcelona, Spain). Culture flasks and dishes were obtained from Falcon (Becton Dickinson BioScience, Le Pont de Claix, France). Melatonin and all other reagents were purchased from Sigma (Sigma- Aldrich, St Louis, MO, USA), unless otherwise indicated.

### Evaluation of cell number and cell death

For MTT assays, cells were seeded onto 96-well plates and the method described by Denizot [20] was followed. Basically, once the treatments were completed, 10 μl of a MTT solution in PBS (5 mg/mL) were added. After 4 hr of incubation at 37°C, one volume of the lysis solution [sodium dodecyl sulphate (SDS) 20% and dimetylformamide pH 4.7, 50%] was added. The mixture was incubated at 37°C overnight and the samples were measured in an automatic microplate reader (μQuant, Bio-Tek Instruments, Inc., Winooski, VT, USA) at the wavelength of 540 nm.

The released lactate dehydrogenase (LDH) was used as an additional index of cytotoxicity. For this assay, cells were seeded onto 24-well plates. Determination of total and released LDH activity was accomplished following specifications of the lactic dehydrogenase based *In Vitro Toxicology Assay Kit* (Sigma-Aldrich, St Louis, MO, USA). Absorbance was determined using an automatic microplate reader (μQuant; Bio-Tek Instruments, Inc., Winooski, VT, USA) at 490 nm.

Trypan blue uptake is indicative of irreversible membrane damage preceding cell death, giving as a result a blue staining in non-viable cells. After treatments cells were harvested and resuspended in 800 μl of PBS and 200 μl of 0.4% trypan blue solution. Three replicates were counted in a Neubauer hemacytometer chamber (Sigma- Aldrich, St Louis, MO, USA). The number of non-stained and stained cells was counted and the percentage of viable and non-viable cells was calculated.

### Flow cytometry

Rhodamine 123 was used to monitor the electrochemical gradient in mitochondria (ΔΨm). Cells were plated in 6-well dishes and, 24 hours later, incubated with Rhodamine 123 (1 μg/ml) in serum-free medium for 20 min at 37°C in the dark. Cells were collected, resuspended in PBS, and incubated with propidium iodide (2 μg/ml) for 10 min at room temperature in the dark. Rhodamine 123 fluorescence of 10,000 live cells per group was measured in a Beckman Coulter FC500 flow cytometer (Beckton Dickinson, Franklin Lakes, NJ, USA).

### Measurement of glucose uptake activity using 2-NBDG

Glucose uptake activity was measured using a fluorescent D-glucose analogue 2-[N-(7-nitrobenz-2-oxa-1,3-diazol-4-yl)amino]-2-deoxy-D-glucose (2-NBDG). Cells were seeded in 96-well plates. After treatment, cells were washed with PBS and incubated with 10 μM 2-NBDG for 35 min. Fluorescence was measured in a microplate fluorimeter FLX-800 (Bio-Tek Instruments, Inc., Winooski, VT, USA) at an excitation wavelength of 467 nm and an emission wavelength of 542 nm.

### Measurement of intracellular lactate levels

Cells were seeded in 150 mm plates and lactate concentration was determined using the *Lactate Assay Kit* (Sigma- Aldrich, St Louis, MO, USA) according to the manufacturer's protocol. Absorbance was measured at 570 nm using an automatic microplate reader (μQuant; Bio-Tek Instruments, Inc., Winooski, VT, USA).

### Colorimetric measurement of glycogen concentration

Glycogen intracellular levels were determined by using the *Glycogen Assay Kit* (Sigma- Aldrich, St Louis, MO, USA) following a modification of the manufacturer´s protocol. Cells were seeded in 150 mm plates, treated as described, collected and homogenized (2x10^6^) in 100 μl of ice-cold water. Reactions were carried out following manufacture´s protocol and absorbance was measured at 570 nm using an automatic microplate reader (μQuant, Bio-Tek Instruments, Inc., Winooski, VT, USA).

### Electron microscopy analysis

After melatonin treatment, cells were fixed with 3% glutaraldehyde (Fluka, Buchs, Switzerland) in 0.1 M phosphate buffer pH 7.3 for 30 min. Cells were then postfixed in 2% w/v OsO_4_ containing 1.25% w/v potassium ferrocyanide, dehydrated in a graded series of acetone solutions and embedded in Spurr resin (*Low viscosity embedding kit*, EMS, Fort Washington, PA, USA). Finally, blocks were polymerized at 70°C for 48 hr. Ultrathin sections (70–90 nm) were obtained using an Ultracut E ultratome (Reichert-Jung, Wien, Austria), stained with 2% p/v uranyl acetate and lead citrate, and photographed with a JEOL 1011 transmission electron microscope (100KV) (Zeiss, Overkochen, Germany) equipped with a system that incorporates a digital photograph camera of 11 Mpixels (Gatan Inc., München, Germany). At least 200 cells on each experimental group were analyzed.

### Determination of ATP levels

Quantitative determination of ATP was carried out by means of the ATP Determination Kit (Molecular Probes, Invitrogen, USA), following manufacturer´s protocol. The assay is based on luciferase's requirement for ATP to produce luminescence. Cells were plated in 6-well plates and lysed in 300 μl of lysis buffer (100 mM potassium phosphate pH 7.2, 2 mM EDTA, 1 mM DTT, 1% v/v Triton) and kept overnight at -20°C. Ten μl of sample were loaded on 96-well white plates, and luminescence was quantified in a microplate luminometer (Thermo Scientific Varioskan Flash, Thermo Fisher Scientific Inc., Waltham, MA, USA). Luminescence was then normalized with sample protein concentration as determined by Bradford assays (*Protein Assay*, BIO-RAD Laboratories, Hercules, CA).

### Western Blot Analysis

For protein expression analysis, cells were seeded on 100 mm plates. After treatments, cells were lysed with ice-cold lysis buffer (150 mM NaCl, 1mM EDTA, 1 mM EGTA, 1% v/v Triton X-100, 2.5 mM sodium pyrophosphate, 1 mM β-glycerophosphate, 1 mM Na_3_VO_4_, 1 μg/mL leupeptin, 2 μg/ml aprotinin, 1 μg/ml pepstatin-A, 110 nM NaF, 1 mM PMSF, 20 mM Tris–HCl pH 7.5). Thirty micrograms of total protein were separated by SDS-polyacrylamide gel electrophoresis and transferred to polyvinylidene difluoride membranes (Amersham Bioscience, Pittsburgh, PA, USA). Blots were incubated overnight at 4°C with appropriate antibodies: hydroxylated-HIF- 1α (1:1000, Cell Signaling, Danvers, MA, USA), phosphorylated AKT (anti-phospho-AKT 1:1000, Cell Signaling, Danvers, MA, USA) and phosphorylated mTOR (anti-phospho-mTOR 1:1000, Cell Signaling, Danvers, MA, USA) as well as glyceraldehyde-3-phosphate dehydrogenase (GAPDH) as a loading control (1:5000, Santa Cruz Biotechnology, Dallas, TX, USA). Immunoreactive polypeptides were visualized using horseradish peroxidase conjugated secondary antibodies (anti-rabbit IgG peroxidase conjugated 1:4000; Santa Cruz Biotechnology, Dallas, TX, USA) and enhanced-chemiluminescence detection reagents (Amersham Bioscience, GE Healthcare Bio-Sciences, Pittsburgh, PA, USA) following manufacturer-supplied protocols. Inmunoblots were analyzed by means of Un-Scan-It Gel 6.0 (Gel graph Digitizing Software-Silk Scientific Corporation, Utah, USA) to provide quantitative values for relative expression of each protein (normalized to its own loading control).

### Data analysis

Results are represented as the average value of at least three independent experiments. Data are represented as the mean +/- SEM. Significance was tested by one-way ANOVA followed by a Student-Newman-Keuls multiple range test. Statistical significance was accepted when P≤ 0.05.

## Results

### Ewing sarcoma cells but not chondrosarcoma cells exhibit features of Warburg effect

**Fig 1:**
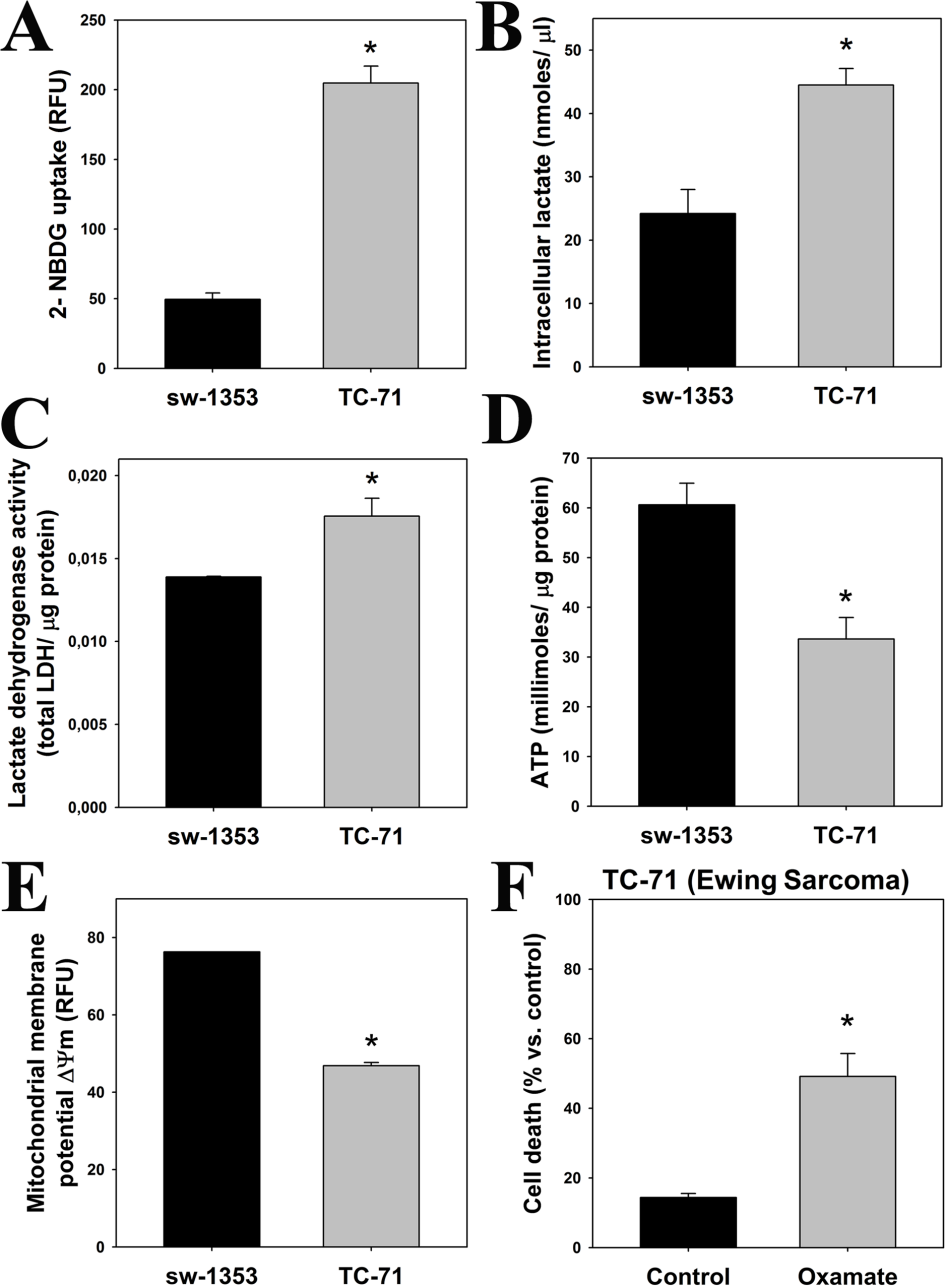
Ewing sarcoma cells exhibit the main hallmarks of Warburg effect. The following parameters indicative of aerobic glycolysis were determined in TC-71 and sw-1353 cell lines (*p≤0.05 vs. sw-1353 cell line, unless otherwise indicated): (A) Glucose uptake (relative fluorescence units, RFU); (B) Intracellular lactate levels (nmoles/μl); (C) LDH activity, normalized versus protein content; (D) ATP levels (millimoles of ATP/μg protein); and (E) Mitochondrial membrane potential (ΔΨm) was expressed as RFUs. (F) TC-71 cells were treated with or without 16.2 mM oxamate for 48 hours, and cell survival rate was determined by means of trypan blue staining. Results were expressed as a percentage of death cells. *p≤0.05 vs. vehicle-treated cells.

Most cancer cells frequently display a metabolic profile different to normal cells. They have higher rates of cytosolic glycolysis, uptaking more glucose and producing an excess of lactic acid [18,21]. We evaluated these parameters characteristic of the tumoral Warburg effect in two sarcoma cell lines: sw-1353 chondrosarcoma cells (which stop proliferating in the presence of melatonin) and TC-71 Ewing sarcoma cells (which are killed by melatonin). As shown in Fig 1A, TC-71 Ewing sarcoma cells consumed higher levels of glucose than sw-1353 chondrosarcoma cells. This is accompanied by an increased lactate production and lactate dehydrogenase (LDH) activity (Fig 1B and 1C), suggesting an increase in fermentative metabolism in TC-71 cells in comparison to sw-1353 cells. TC-71 cells also exhibited lower levels of ATP (Fig 1D) and a less significant mitochondrial membrane potential (ΔΨm) than sw-1353 cells (Fig 1E). Inhibition of LDH by oxamate in the TC-71 cell line caused cell death, indicating that this metabolic pathway is essential for their survival (Fig 1F). All results together show an evident difference in basal energy metabolism between chondrosarcoma and Ewing sarcoma cells, which in turn may be related to the differential effect of melatonin on both cell types. In those cells where we found Warburg effect (TC-71) melatonin induces cell death [8,10], whereas in those cells that present “normal” glycolytic metabolism with little or no participation of the aerobic glycolysis (sw-1353) melatonin inhibits cell proliferation [10].

### Opposite effects of melatonin in glucose metabolism in Ewing sarcoma and chondrosarcoma cells

**Fig 2:**
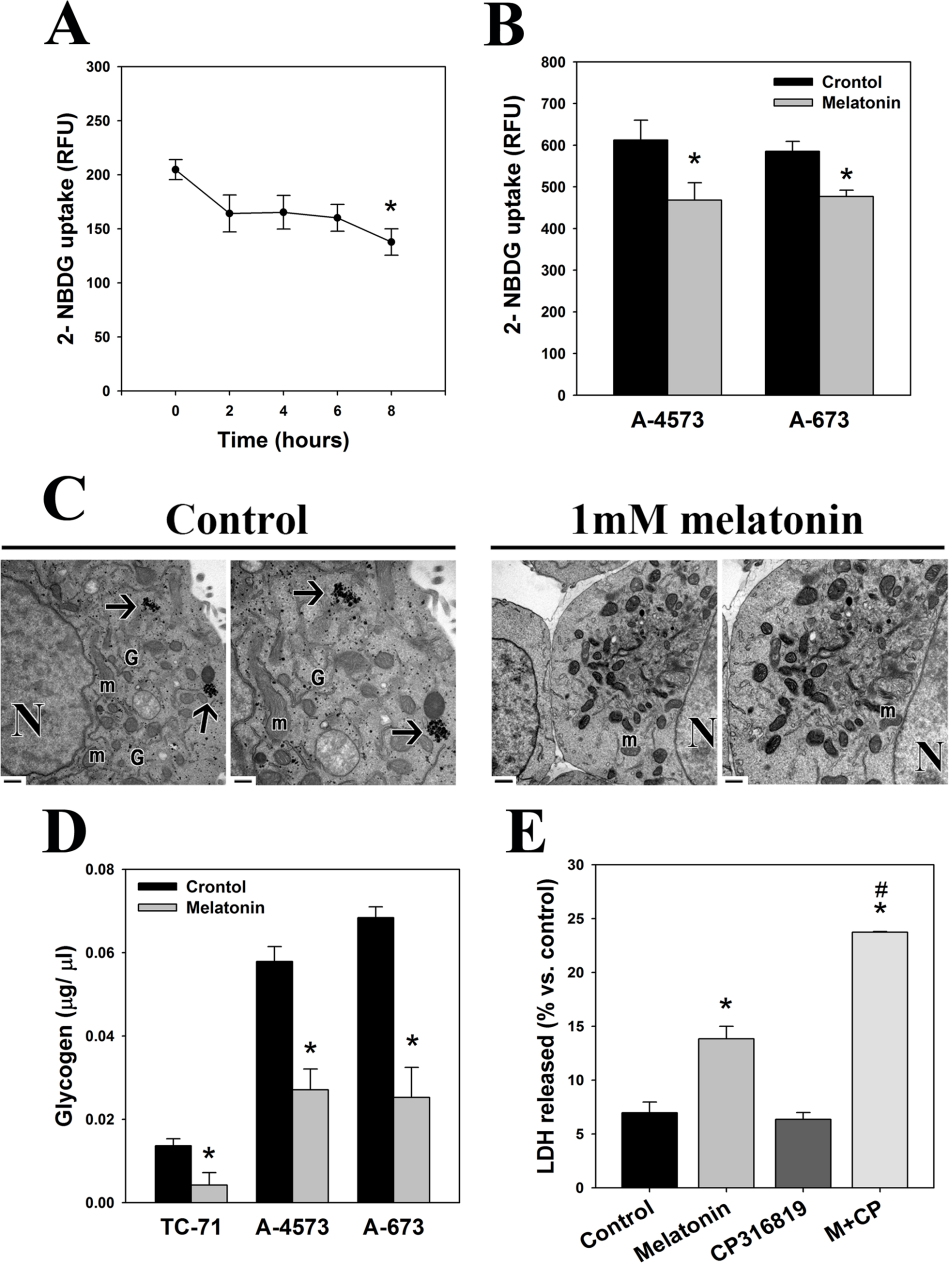
Melatonin inhibits glucose uptake and depletes glycogen stores in Ewing sarcoma cells. (A) Glucose uptake was measured in TC-71 cells treated with o without 1 mM melatonin for several times (2h, 4h, 6h and 8h). Data are expressed as RFU (relative fluorescence units). (B) Glucose uptake was measured in A4573 and A-673 cells treated with o without 1 mM melatonin for 8h. Data are expressed as RFU (relative fluorescence units). (C) Electron microscopy images showing the decrease in glycogen stores after treatment of Ewing sarcoma cells with 1 mM melatonin for 24 hours. Black arrows indicate glycogen stores that appear at cytoplasm forming rosettes. Also dense granules of glycogen can be observed free in the cytoplasm in control groups. N, nucleus; m, mitochondria; G, golgi apparatus areas. Right images on each experimental group correspond to a higher magnification of left image. Bars: 1μm (left image) /0.5μm (right image). (D) Intracellular glycogen levels were assessed in TC-71, A-4573 and A-673 cells after incubation with 1 mM melatonin for 4 hours. (E) Cell death was determined by means of the LDH-release assay. Ewing sarcoma cells were treated with or without 1 mM melatonin and 10 μM CP316819 for 72 hours, and cell death was calculated as the ratio between released and total LDH activity on each experimental group. Data are expressed as the percentage of control (vehicle-treated cells).*p≤0.05 vs. vehicle-treated cells; #p≤0.05 vs. melatonin-treated cells.

In order to identify the possible effect of melatonin in aerobic glycolysis we studied first glucose uptake in the presence or absence of the hormone. Melatonin induced a rapid decrease in glucose uptake in TC-71 Ewing sarcoma cells significant after 8 hours of treatment (Fig 2A). This decrease in glucose uptake after 8 hours of treatment was observed also in two other Ewing sarcoma cell lines, A-4573 and A-673 (Fig 2B). Decreased glucose availability could force TC-71 cells to use intracellular glucose stores (glycogen) in order to obtain energy. Melatonin actually abolished glycogen reservoirs that were easily observed by electron microscopy in control groups both as dense granules free in the cytoplasmic matrix and forming rosettes (Fig 2C). Biochemical determination of glycogen levels confirmed a drastic decrease in glycogen levels after 4 hours not only in TC-71 cells but also in A-4573 and A-673 cells (Fig 2D). Moreover, CP316819, a specific inhibitor of glycogen breakdown, strongly enhanced melatonin-induced cell death in TC-71 cells (Fig 2E), indicating that normal glycogen breakdown was essential for cell viability.

**Fig 3:**
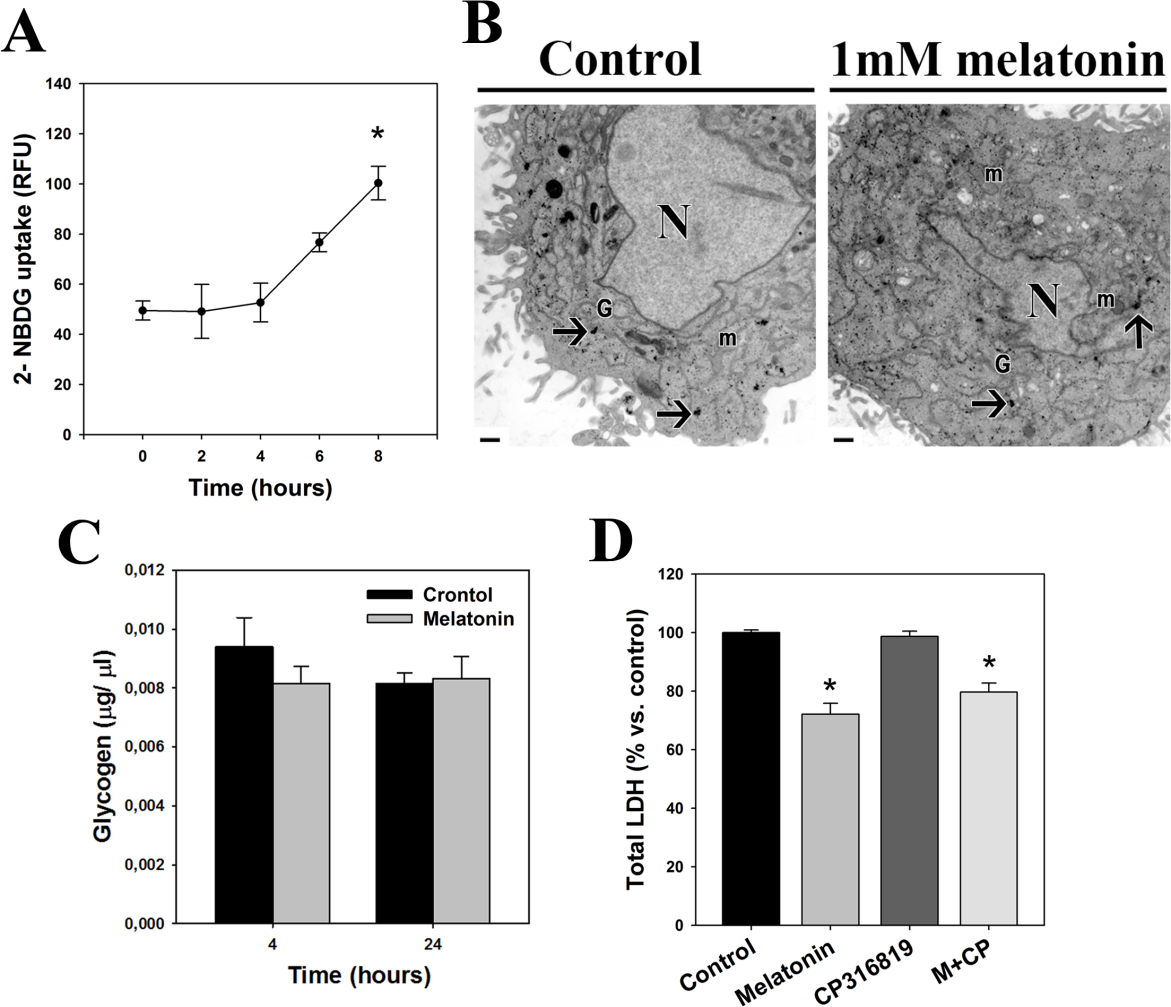
Melatonin does not inhibit aerobic glycolysis in chondrosarcoma cells. (A) Glucose uptake was determined in sw-1353 chondrosarcoma cells treated with o without 1 mM melatonin for several times (2h, 4h, 6h and 8h). Data are expressed as RFU (relative fluorescence units). (B) Melatonin effects on glycogen stores were evaluated in sw-1353 cells incubated with 1 mM melatonin for 24 hours, by means of electron microscopy. Black arrows indicate glycogen stores that appear at cytoplasm forming rosettes. Also dense granules of glycogen can be observed free in the cytoplasm in both control and melatonin treated groups. N, nucleus; m, mitochondria; G, golgi apparatus areas. Bars: 0.5μm. (C) Intracellular glycogen levels were assessed in sw-1353 cells after incubation with 1 mM melatonin for 4 and 24 hours. (D) Chondrosarcoma cells were treated with or without 1 mM melatonin and 10 μM CP316819 for 72 hours, and cell number was determined by the quantification of total LDH present in cell culture. Data are expressed as the percentage of control (vehicle-treated cells). *p≤0.05 vs. vehicle-treated cells.

In contrast, melatonin induced an increase in glucose uptake in sw-1353 chondrosarcoma cells (Fig 3A) and, consistently, it did not induce glycogen breakdown (Fig 3B and 3C). In addition, CP316819 did not alter melatonin antiproliferative effects (Fig 3D).

### Melatonin inhibits fermentative metabolism in Ewing sarcoma cells but not in chondrosarcoma cells

**Fig 4:**
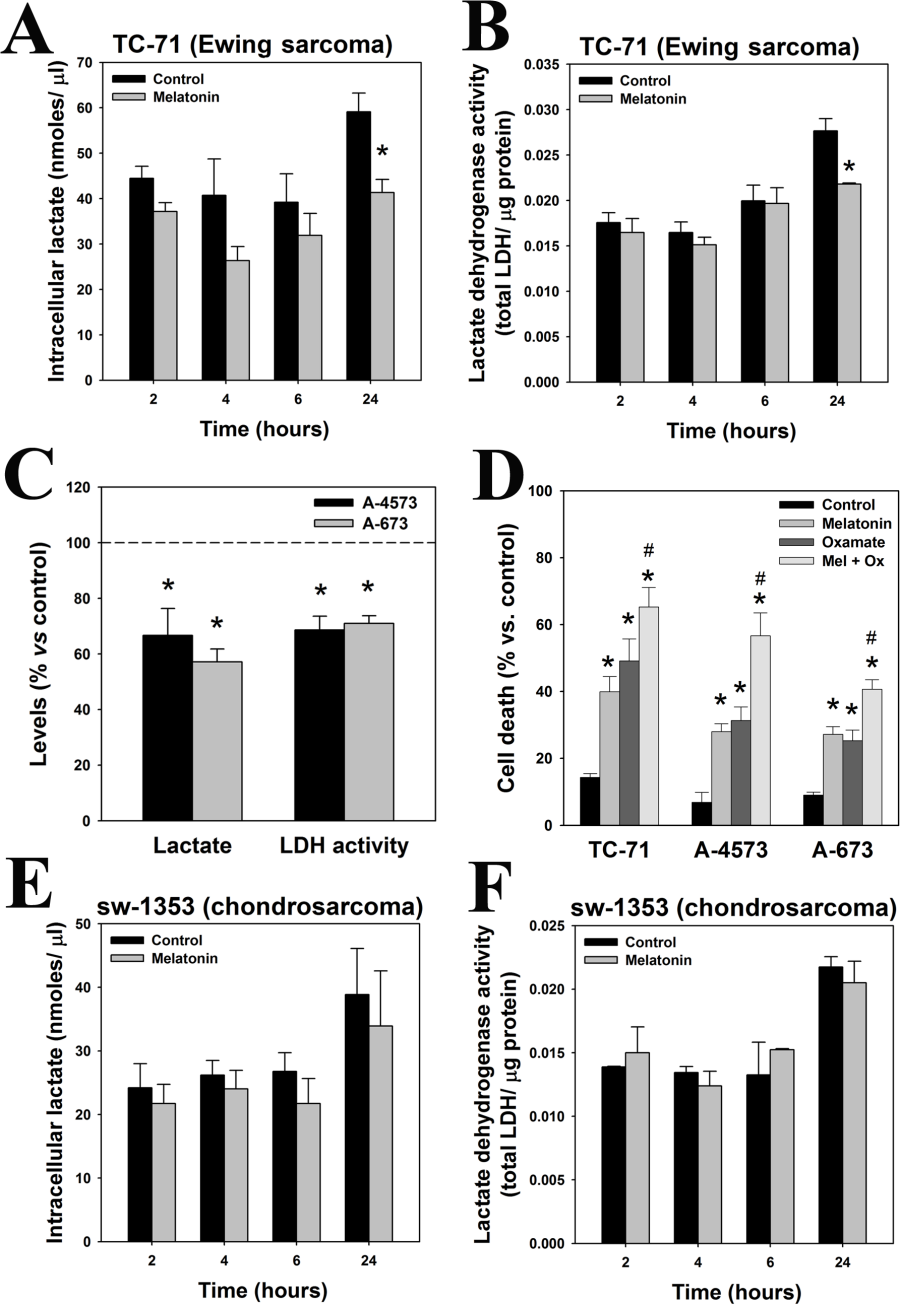
Aerobic glycolisis is inhibited by melatonin in Ewing sarcoma cells. (A) Lactate levels (nmoles/μl) were quantified in TC-71 cells treated with 1 mM melatonin for 2, 4, 6 and 24 hours. (B) LDH activity was evaluated in TC-71 cells incubated with 1 mM melatonin for several times (2h, 4h, 6h and 24 hours), and results were normalized with protein content (μg) in each sample. (C) Intracellular lactate levels and LDH activity were evaluated in A-4573 and A-673 Ewing sarcoma cells treated with 1 mM melatonin for 24 hours. Data are represented as percentage versus control group. (D) Cell viability (% of death cells) was evaluated by trypan blue after the incubation of TC-71, A4573 and A-673 Ewing sarcoma cells with 1mM melatonin and 16.2 mM oxamate for 48 hours. (E) Intracellular lactate levels (nmoles/μl) were quantified in sw-1353 chondrosarcoma cells treated with 1 mM melatonin for 2, 4, 6 and 24 hours. (F) LDH activity was evaluated in sw-1353 chondrosarcoma cells incubated with 1 mM melatonin for 2, 4, 6 and 24 hours, and results were normalized with protein content (μg) in each sample. *p≤0.05 vs. vehicle-treated cells; #p≤0.05 vs. melatonin-treated cells.

A hallmark of the Warburg effect is that pyruvate is reduced to lactate instead of entering the Krebs cycle and undergoing oxidative phosphorylation (OXPHOS) [22]. Our data shows that intracellular lactate levels (Fig 4A) and LDH activity (Fig 4B) were significantly reduced in TC-71 cells after treatment with melatonin for 24 hours. The inhibitory effect in LDH activity and lactate production is not exclusive from TC-71 cells but extended to A-4573 and A-673 Ewing sarcoma cell lines (Fig 4C). Moreover, the LDH inhibitor oxamate significantly enhanced melatonin toxicity in the three Ewing sarcoma cell lines (Fig 4D). These data indicate that fermentative metabolism is essential for Ewing sarcoma cells survival and its blockage by melatonin, oxamate or both is lethal.

In contrast to Ewing sarcoma, melatonin did not induce changes in intracellular lactate levels or LDH activity in sw-1353 chondrosarcoma cells (Fig 4E and 4F). Consistently, oxamate was not toxic neither potentiated melatonin antiproliferative effect (data not shown). It seems that chondrosarcoma cells are preferentially using another energy pathway different from fermentative metabolism, which is not regulated by melatonin.

**Fig 5:**
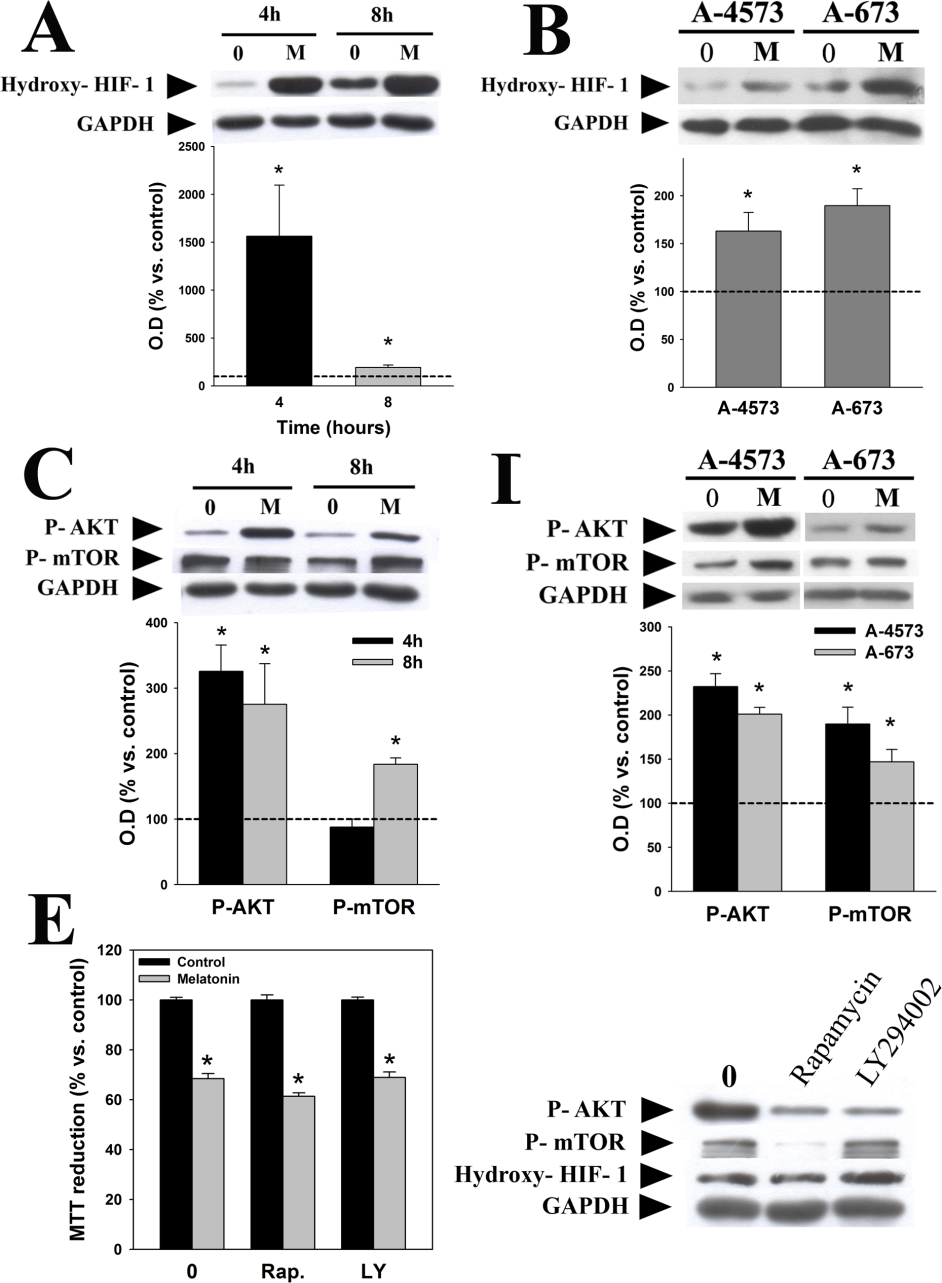
Melatonin inhibits HIF-1α in a PI3K/AKT/mTOR independent pathway. Western blot analyses were carried out to identify the effect of melatonin (1 mM, 4 and 8 hours) on the activation of HIF-1α in TC-71 cells (A) and A-4573 and A-673 (1mM, 4 hours) cells (B). AKT and mTOR activation in TC-71 (C) and A-4573 and A-673 cells (D) was evaluated using specific phosphoantibodies. GAPDH was used as a loading control in all cases. A representative blot is showed. Optical density of bands was measured and values of the hydroxy-HIF-1α (inactivated form), p-AKT or p-mTOR bands were normalized versus GAPDH. Results are represented as percentage of the values found in vehicle-treated cells (dotted line). (E) *Left panel*, cell viability was evaluated by MTT reduction assay after treatment of TC-71 cells with 1 mM melatonin alone or in combination with 10 nM rapamycin or 10 μM LY294002 for 48 hours. Data are expressed as the percentage of vehicle-treated cells. *Right panel*, Representative western blot showing the relative protein level of p-AKT, p-mTOR and hydroxy-HIF-1α after 10 nM rapamycin or 10 μM LY294002 treatment during 24 hours in TC-71 cell line. p*≤0.05 vs. vehicle-treated cells.

Activation of the master regulator of oxygen homeostasis HIF-1α is essential for maintenance of Warburg effect [23]. As shown in Fig 5, melatonin induced a significant increase in the inactivated (hydroxylated) form of HIF-1α in TC-71 (Fig 5A), A-4573 and A-673 cells (Fig 5B), which is consistent with its inhibition of Warburg effect. PI3K/AKT/mTOR signaling can increase HIF-1α activity by inducing its stabilization, and we and others have shown that melatonin regulates the PI3K/AKT/mTOR pathway in some tumor types [5,24]. However, melatonin activated AKT and mTOR in TC-71 (Fig 5C), A-4573 and A-673 cells (Fig 5D), which is not consistent with the inactivation of HIF-1α. On the other hand, rapamycin or LY294002, specific inhibitors of mTOR and PI3K respectively, did not prevent melatonin-induced decrease in TC-71 cell viability (Fig 5E, *left panel*). Both inhibitors reduced the phosphorylation of their target proteins, as expected, and none of them modified HIF-1α expression (Fig 5E, *right panel*). These data suggest that PI3K/AKT/mTOR activation may not be responsible for HIF-1α regulation by melatonin in Ewing sarcoma cells, and therefore this pathway is probably not involved in melatonin toxicity.

## Discussion

Melatonin proapoptotic effect in some cancer cell types has been extensively studied in recent years, but the exact mechanism remains unknown. We show for the first time that melatonin cytotoxic effect is related to an inhibition of Warburg effect in Ewing sarcoma cells.

Reprogramming of energy metabolism is a capability involved in the pathogenesis of most tumors, and has become one of the main hallmarks of cancer. This theory relies on previous research conducted by Otto Warburg [18], who hypothesized that most tumor cells obtain energy mainly by transformation of glucose to lactate rather than oxidizing pyruvate into the mitochondria. This process, known as Warburg effect or aerobic glycolysis, is triggered by alterations in signaling pathways involved in glucose uptake and metabolism, which in turn can also regulate mitochondrial metabolism [25]. The mitochondrial electron transport chain and OXPHOS are the main sources of cellular ROS and, therefore, alterations in mitochondrial metabolism could have consequences for the intracellular REDOX state of tumoral cells.

Melatonin is a well-known antioxidant and has various effects at mitochondrial level and, most importantly, we and others have shown that its antitumoral effects are related with its regulation of the intracellular REDOX state. Thus, inhibition of cell proliferation correlates with a decrease in intracellular ROS, while melatonin cytotoxic effects are associated with an increase in oxidative stress [10]. The results presented here indicate that the different effects of melatonin could be related to differences in the metabolic pattern of cancer cells.

Ewing sarcoma cells show an increased basal glucose uptake, higher levels of intracellular lactate and LDH activity, lower ATP production and decreased mitochondrial functionality in comparison to chondrosarcoma cells. Inhibition of LDH activity kills cancer cells that are metabolically dependent on the Warburg effect [26] and, consistently, oxamate kills Ewing sarcoma cells but not chondrosarcoma cells. These results strongly suggest that the metabolism of TC-71 cells, but not chondrosarcoma cells, relies on aerobic glycolysis and the Warburg effect.

Our results also suggest that melatonin inhibits glycolytic metabolism of Ewing sarcoma cells but not of chondrosarcoma cells. It induces a decrease in glucose uptake, lactate levels and LDH activity, and enhances oxamate cytotoxic effect, further confirming that aerobic glycolysis is essential for the survival of Ewing sarcoma cells. Besides external supply, cells can also obtain glucose from the degradation of glycogen, the major intracellular glucose storage. Thus, melatonin´s inhibition of glucose uptake could cause the breakdown of glycogen stores observed in Ewing sarcoma cells, possibly due to an attempt to obtain energy and maintain cell viability. This is confirmed by the fact that blockage of glycogen breakdown enhances the toxicity of melatonin and is highly lethal for these cells. Inhibition of glycogen phosphorylase- enzyme that breaks down glycogen into glucose subunits- triggers apoptotic cell death due to the absence of energetic substrate in some tumor types [27], like melatonin does. Blockage of glycolytic metabolism currently constitutes a major target to prevent cancer growth, with various experimental treatments being already tested in preclinical studies. These include genetic or pharmacological inhibition of glycolytic enzymes or LDH [28–30]; or the use of non-metabolizable analogues, such as 2-Deoxyglucose (2-DG) [31; 32] or 3-Bromopyruvate [33]. Many of these treatments prevent tumor progression, induce an increase in ROS production and kill cancer cells [30], again consistent with melatonin effects in Ewing sarcoma cells.

Melatonin inactivates HIF-1α in Ewing sarcoma cells, which could account for the decrease in aerobic glycolysis. This transcription factor is primarily responsible for the increase in glycolytic activity in most cancer cells, allowing them to survive. Its activation is essential for cancer cells to exhibit Warburg effect, since it increases the activity of the vast majority of the enzymes involved in aerobic glycolysis even under normoxic conditions [23]. Inhibition of the transcription factor HIF-1α has been repeatedly suggested as a therapeutic target against cancer, because of its key role in the Warburg effect could allow fine control of tumor growth [34]. In this sense melatonin has been described to decrease invasion and cell migration by blocking HIF-1α in some tumor types [35]. Furthermore, in hypoxic environments, melatonin is able to decrease HIF-1α expression levels in several tumor types [36]. Finally, in vivo antitumoral and anti-angiogenic effects of melatonin have been also suggested to be mediated by a decrease in HIF-1α expression [37]. Although there are a number of studies relating the activation of PI3K/AKT/mTOR pathway with the activation of HIF-1α [38,39], and melatonin regulates the PI3K/AKT/mTOR pathway in several models [5,24], our results show that these events are not connected in Ewing sarcoma cells.

Taken together, the data presented in this work demonstrate the ability of melatonin to regulate aerobic glycolysis or Warburg effect in cells highly dependent on this metabolic pathway, such as Ewing sarcoma cells. This effect is closely associated with its toxicity in these cells. Given that fermentative metabolism is currently considered a possible therapeutic target in the fight against cancer, we should consider melatonin, alone or in combination with other antitumoral agents, as a possible tool deserving further attention. Identifying the intracellular mechanisms by which melatonin is able to control this metabolism is of special interest to understand why some tumor types are sensitive to its antitumoral effects while others are not. Such understanding could lay the groundwork for the development of new personalized therapeutic strategies, based on a match between specific drugs and the intrinsic features of each cancer. Thus, the relevance of our findings stems from the fact that melatonin could be used most efficiently as a personalized antitumoral agent, if we were able to determine the metabolic profile of each tumor in patients in vivo or in biopsies of their tumors.
